# Supplementary material for: System-Specific Epigenetic Aging Signatures in Autistic Adults
Source: bioRxiv. 2026 Sep 18:2026.09.12.751174. Preprint. [Version 1] doi: 10.64898/2026.09.12.751174 (PMC13596294; doi:10.64898/2026.09.12.751174)
Supplement: Supplementary file 2 [file media-14.docx]

**Supplementary Figure 1.** Leave-one-dataset-out (LODO) analyses for Brain, Blood, Liver, and Metabolic subscores. Left: Residualized scores for the autistic group (red) and each individual control dataset (blue). Right: Modelled group differences between autistic and control participants (β, 95% CI) after sequentially excluding each control dataset. The axis labels indicate which control dataset was excluded from the model; “None” represents the primary model reported in the main analyses, in which all control datasets were included.
